# The prevalence and nature of the use of preconception services by women with chronic health conditions: an integrative review

**DOI:** 10.1186/s12905-015-0165-6

**Published:** 2015-02-18

**Authors:** Amie Steel, Jayne Lucke, Jon Adams

**Affiliations:** 1 Office of Research, Endeavour College of Natural Health, Brisbane, Queensland Australia; 2 Australian Research Centre in Complementary and Integrative Medicine, University of Technology Sydney, Ultimo, New South Wales Australia; 3 Australian Research Centre in Sex, Health and Society, Faculty of Health Sciences, La Trobe University, Melbourne, Victoria Australia; 4 UQ Centre for Clinical Research, The University of Queensland, Brisbane, Queensland Australia

**Keywords:** Preconception care, Chronic disease, Women’s health services

## Abstract

**Background:**

There is growing evidence that preconception care may have an important role in preventing short and long term adverse health consequences for women and their offspring. This is particularly the case for women with chronic health conditions due to the rising prevalence of chronic disease in global populations. With this in mind, this paper presents an integrative systematic review of contemporary research outlining the use of preconception services and practices by women with chronic health conditions.

**Methods:**

A search was conducted through PubMed, CINAHL, AMED, and Maternity and Infant Care databases which identified 672 papers examining preconception care and preconception services for women with chronic health conditions. Fourteen papers which were written in English, presented original research, and reported on the prevalence or nature of use of preconception care by women with chronic health conditions were included in the review. Critical appraisal of study quality and thematic categorical grouping of identified papers was undertaken.

**Results:**

Current research evidence, as identified through this review, examines three major topic areas: the prevalence of preconception care practices, use of services and characteristics of users; knowledge of the value and impact of preconception care and availability of preconception services for women with chronic health conditions; and women’s attitudes, approaches and experiences of preconception care and preconception services. Prevalence estimates of engagement with preconception care range between 18.1% and 45%, with most studies focusing on women with type 1 or 2 diabetes. Significant gaps in women’s knowledge of preconception care for women with chronic health conditions were also identified. Women with chronic health conditions reported experiencing emotional distress as a result of their engagement with preconception care services. They also commonly described feeling a need to employ discipline to comply with preconception care programs, and experiencing a fear of pregnancy complications.

**Conclusion:**

Future research requires a broad and sophisticated approach to research design and analysis, improved consideration of temporal changes to women’s health behaviour, representative samples to more effectively inform health policy, and a deeper understanding of women’s motivations, attitudes and perceptions of preconception care to assist in the development of tailored preconception health services.

## Background

Preconception care is an approach to health promotion and preventive medicine which focuses on interventions that identify and modify biomedical, behavioural and social risks to a woman’s health or pregnancy outcome [1]. By its nature, preconception care relates to care before pregnancy, whether it be a first pregnancy or between consecutive pregnancies, and the importance of this component of contemporary health care has been acknowledged by a range of international bodies and organisations representing health professionals and policy makers [2-5].

### Impact of preconception health status on outcomes for women and neonates

Preconception care has received increased attention due to growing evidence that maternal health prior to conception can directly affect the health of the mother and the fetal environment during pregnancy [2]. The majority of research attention over the last 20 years has been directed to the benefits of folic acid supplementation in preventing birth defects [6-10]. The broader field of preconception care research emphasises the impact of the fetal environment on adverse outcomes such as miscarriage [11], stillbirth [12], congenital disorders [13], and macrosomia [14]. Fetal environment has also been found to impact on risk of the development of chronic diseases such as obesity [15], diabetes and cardiovascular disease [16], and cancer [17] through epigenetic and other cellular responses to developmental exposures [18]. Maternal health behaviours which have been clearly identified as important in the context of preconception care include diet [19], smoking [20], alcohol consumption [21] and exposure to communicable diseases [22].

In light of these developments, and the higher prevalence of important risk behaviours in sub-groups including women with lower socio-economic status [21,23] and teenagers [24], attempts have been made to inform women of childbearing age and modify preconception health behaviours through the development of health policy and guidelines alongside the implementation of preconception care programs within the community [25-29].

### Importance of preconception care for women with chronic health conditions

Chronic and non-communicable diseases are the largest cause of death in the world and as such have a well-established global epidemiological and economic impact [30]. In addition, the prevalence of the most common chronic diseases is growing, particularly in developing countries, and is projected to continue to increase [30]. This has led to calls for key stakeholders, including public health researchers, to direct more research towards chronic disease and its impact [30]. In line with this, the clinical impact of preconception care for women with chronic health conditions has received focused attention from researchers. Diabetes has received particular attention because the preconception health of women with either type 1 or type 2 diabetes has been found to impact on the risk of congenital malformations, preterm delivery and perinatal mortality of their offspring [31]. Pre-pregnancy maternal obesity is linked to an increased likelihood of preterm birth and low birth weight [32] as is hypertension [33], depression [34] and autoimmune disease [35]. Whilst some research has reported no adverse effects from non-immune hypothyroidism in the preconception period if appropriately managed through antepartum care [36], untreated thyroid dysfunction is also linked with an increased risk of obstetric and neonatal complications [37]. In contrast, the risk of adverse pregnancy outcomes for women with epilepsy is directly associated with the antiepileptic drugs used as part of treatment [38].

Some commentators and policymakers have attempted to inform the development of preconception programs to improve prenatal health status and reduce the incidence of adverse outcomes in offspring of women with chronic health conditions [39-41]. Preconception programmes and interventions have been developed and implemented for women of childbearing age with chronic health conditions with varying degrees of success [31,42-45]. A recent systematic review and meta-analysis examining the impact of preconception care for preventing and managing chronic disease confirmed the value in preconception interventions [45]. In particular, the review concluded a reduced incidence of congenital malformations for preconception care of women with diabetes as well as highlighting the importance of preconception management of epilepsy [45].

Effective intervention programmes requires not only knowledge of the effectiveness of the intervention but also insights into the knowledge, attitudes, and health behaviours of the target populations. It is also important that those responsible for preconception programmes and interventions have an understanding of the characteristics which influence women’s likelihood to engage with preconception services or undertake health preconception practices. Insights beyond effectiveness outcomes such as these have the capacity to enhance preconception service delivery by ensuring it is tailored and responsive to the needs of the community. In answer, this paper provides a review of international contemporary literature with a specific focus upon the prevalence and nature of use of preconception care practices and services by women with chronic health conditions. By focusing on these factors this review offers an important supplement to efficacy and effectiveness research and will be able to inform future preconception care researchers as well as clinicians and policy makers.

## Methods

### Search methods

A database search was conducted to identify peer-reviewed literature published between 2003 and October 2014 examining the use of preconception care practices and services by women with a chronic health condition. Literature published before 2003 were not included as previous research examining women’s attitudes and knowledge towards preconception care has identified rapid changes to the women’s understanding of preconception care over time [46]. Based upon this, manuscripts more than 10 years old would hold limited value to future work. The search was conducted in November 2014 and included PubMed, CINAHL, AMED, and Maternity and Infant Care. The search terms included: *preconception care* or *prenatal care*; *thyroid diseases*; *diabetes mellitus*; *cardiovascular diseases*; *depressive disorder*; *obesity*; *allergy*; *immunology*; and *chronic disease*. Manual searching of the reference lists of identified papers was also conducted to ensure no relevant papers had been overlooked. Papers were included if they were written in English, presented original research, and reported on the prevalence or nature of use of preconception care. Manuscripts were excluded if they presented results from experimental research designs, or only reported on the impact of preconception care on pregnancy or birth outcomes.

### Search outcome

**Figure 1:**
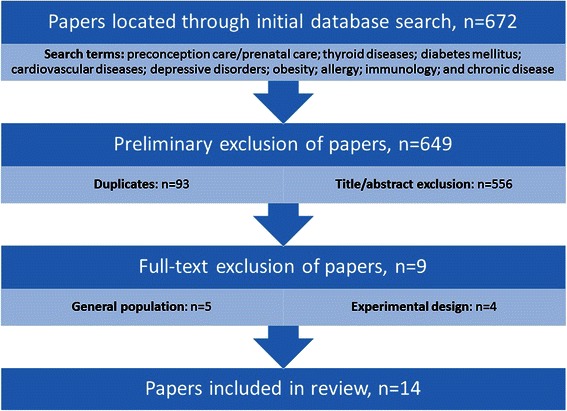
**The literature search and selection process (figure uploaded separately).**

The search results (n = 672) were imported into Endnote referencing and bibliography management software. A total of 93 papers were excluded as duplicates and an additional 556 papers were identified as not complying with the inclusion criteria based upon their title or abstract. Upon reviewing the full text articles, 5 articles were excluded due to their focus on the general population rather than women with a chronic health condition and 4 other papers were excluded as they reported an experimental design. The process undertaken for this review is presented in Figure 1.

### Analysis of included papers

#### Critical appraisal and analysis

The critical appraisal of study quality for research examining clinical outcomes was conducted by applying a quality scoring system, modified from a system previously developed and applied [47,48]. This system was designed to systematically compare and evaluate the studies reviewed and allow for appraisal across three dimensions: methodology; reporting of participants’ characteristics; and preconception care services examined. Methodology was appraised according to the use of a representative sampling strategy, adequate sample size, a response or participation rate of >75%, and low retrospective measurement error (defined as prospective data collection or retrospective data collection within the previous 12 months) [47]. Quantitative papers were appraised according to a determination of sample size based upon a power analysis of >80%. Where the power analysis was not reported a sample of >385 was accepted based upon standard precision analysis principles to account for possible sampling error [49]. Qualitative papers were appraised according to whether they reported achieving thematic saturation during data collection [50]. Appraisal of sample size for qualitative studies was based upon a minimum sample of 15 participants where thematic saturation was not reported. Finally, the studies were assessed for the examination of preconception care services. Studies were critiqued based upon their examination of comprehensive preconception clinical care services – here defined as services where multiple components of preconception care are delivered [51] - rather than uptake of a stand-alone health behaviour, as well as preconception care-related health promotion and patient education. The three dimensions which form the quality assessment tool were selected for inclusion in the critical framework in line with the aim of this review. Each component of the three dimensions was awarded 1 point if the paper achieved the minimum defined requirement and cumulative scores for each paper were calculated with a maximum potential score of 11. Scores for the studies were assigned independently by two authors. The results were then compared and differences resolved by discussion. A study receiving a quality score of >8 was determined to be of acceptable quality as it reflects significant representation across at least 2 of the 3 domains of interest: *methodology*, *reporting of participant characteristics*, and *preconception services examined*.

Categorical grouping of the identified papers was also undertaken by the first author through an inductive process grounded in the content of the papers. This iterative process involved reading and re-reading the papers, extracting relevant information, and identifying common themes within the reported findings. Upon developing the categories, the included studies were examined again and assigned to a relevant category. Individual studies were assigned to as many categories as appropriate based upon the content of the paper.

## Results

An overview of all papers included in the review including preliminary categorical analysis is outlined in Table 1. The review papers identified 14 studies conducted in North America [52-57], the United Kingdom [58-61], Australia [62,63], France [64], and Malta [65]. The studies were published between 2005 and 2014 with the majority [53,56-61,63,65,66] published since 2010. The research designs varied between the studies with both quantitative [52,55-57,61,63-65] and qualitative [53,54,58-60,62] methodologies reported. The quantitative studies utilised a number of survey design approaches and drew on sample sizes between 27 [65] and 6385 [52]. The qualitative studies employed primarily semi-structured interviews involving between 7 [62] and 29 [60] women, although one study used focus groups conducted with a total of 72 women [53]. All of the identified papers examined women with type 1 diabetes [52-66], however women with type 2 diabetes [52,53,56-58,60,61,63], hypertension [53,57] and overweight/obesity [52,53] were also explored in some studies.

**Table 1:** **Summary of identified papers examining preconception care in women with chronic health conditions with thematic categorical groupings**

Author (Year)	Country	Method	Data source/Participant recruitment	Target population	Conditions examined	Sample	Theme		
							I	II	III
Case et al. [52]	United States	Cross-sectional survey	Texas 1999–2003 Behavioural Risk Factor Surveillance System	Non-pregnant women	Obesity, overweight, diabetes	6385	X		
Chuang et al. [53]	United States	Focus groups	Community and outpatient clinic	Non-pregnant women	Diabetes, hypertension, obesity	72		X	
Diabetes and Pregnancy Group [64]	France	Cross-sectional survey	Patients (outpatients and hospitalised) from specialist diabetes centres (n = 11)	Non-pregnant women	Type 1 diabetes	138		X	
Griffiths et al. [54]	United Kingdom	Semi-structured interviews	UK specialist diabetes antenatal clinics (n = 4)	Pregnant women	Type 1 diabetes	15		X	X
Kallas-Koeman et al. [56]	Canada	Prospective observation and survey	Electronic medical records from diabetes clinic	Non-pregnant women	Type 1 and Type 2 diabetes	464	X		
Kim et al. [55]	United States	Prospective survey	Translating Research into Action for Diabetes (TRIAD) cohort study	Non-pregnant women	Type 1 diabetes	302	X		
King and Wellard [62]	Australia	Collective case study (via in-depth interviews)	Local diabetes services and youth diabetes website	Women with previous pregnancy	Type 1 diabetes	7			X
Lavender et al. [58]	England	Semi-structured interviews and focus groups	Obstetric and diabetes clinics in hospitals in North-West England (n = 3)	Women with previous pregnancy	Type 1 and Type 2 diabetes	22			X
McCorry et al. [59]	Ireland	Semi-structured interviews		Non-pregnant women	Type 1 diabetes	14			X
Mittal et al. [57]	United States	Cross-sectional survey	San Francisco General Hospital Family Health Center registry	Non-pregnant women	Diabetes, hypertension, obesity	27		X	X
Murphy et al. [60]	England	Semi-structured interviews	Diabetes specialist antenatal clinics (n = 3)	Women who did not attend preconception care	Type 1 or Type 2 diabetes	29			X
Sapiano et al. [65]	Malta	Longitudinal survey	Diabetes hospital outpatient clinic	Non-pregnant women	Type 1 diabetes	27		X	
Tripathi et al. [61]	England	Cross-sectional survey	Northern Diabetes in Pregnancy Survey	Women with previous pregnancy	Type 1 or Type 2 diabetes	588	X		
Zhu et al. [63]	Australia	Prospective survey	Diabetes hospital outpatient clinic	Non-pregnant women	Type 1 or Type 2 diabetes	51	X	X	

I: Prevalence of engagement with preconception services and characteristics of users.

II: Women’s knowledge of preconception care benefits, preconception information sources, and availability of preconception services.

III: Women’s attitudes and experiences of preconception care and preconception services.

The quality score of each relevant individual study is reported in Table 2. The quality appraisal of included papers identified only 4 papers with a score of 8 [52,53,55,61]. Many studies did not report sufficient detail on the response or participation rate [52-54,56-65] and the majority did not report the time since participant’s diagnosis of the chronic health condition [52-54,57,61-63,65] but beyond this the quality issues were inconsistent across studies. Only two studies did not address any of the criteria for methodological quality [62,64].

**Table 2:** **Quality assessment of manuscripts identified through the literature review process BIBU**

	Dimensions for quality assessment	Lavender et al.[58]	Case et al.[52]	Murphy et al.[60]	Diabetes & Pregnancy Group[64]	McCorry et al.[59]	Tripathi et al.[61]	Zhu et al.[63]	Mittal et al.[57]	Chuang et al.[53]	Kallas-Koeman et al.[56]	Kim et al.[55]	Sapiano et al.[65]	Griffiths et al.[54]	King and Wellard[62]
**Methodology**	Representative sampling strategy		x				x		x			x			
	Sample size^≠^	x	x	x			x			x	x			x	
	Response/participation rate >75%						x					x			
	Low retrospective measurement bias		x			x	x	x		x	x		x	x	
**Reporting of participant characteristics**	Pregnancy status	x	x	x	x	x	x	x	x	x	x			x	
	Age	x	x	x	x	x	x	x	x	x	x	x	x	x	x
	Ethnicity	x	x	x			x		x	x		x			
	Indicator of socioeconomic status		x		x			x		x		x			
	Duration of chronic health condition	x		x	x	x					x	x			
**Preconception services examined**	Preconception clinical care delivery	x		x	x	x	x	x		x	x	x	x	x	x
	Health promotion and patient education	x	x	x	x	x			x	x		x	x	x	x
**Total quality score**		**7**	**8**	**7**	**6**	**6**	**8**	**5**	**5**	**8**	**6**	**8**	**4**	**6**	**3**

^≠^Sample size defined in quantitative studies as >500 or with a determined statistical power of >80% and in qualitative studies as >15 or reported thematic saturation.

Through analysis of the findings reported in each of the identified studies, three descriptive categories of results was developed: prevalence of preconception care practices, use of preconception services and characteristics of users; knowledge of the value and impact of preconception care and availability of preconception services; and attitudes, approaches and experiences of preconception care and preconception services.

### Prevalence of preconception care practices, use of preconception services, and characteristics of users

Prevalence of engagement with preconception care services and practices, and the characteristics of women who undertook to use preconception care was examined in 5 of the reviewed papers [52,55,56,61,63]. The prevalence estimates ranged between 18.1% through to 45% depending upon the health condition being examined [52,56,61,63]. Primarily, the studies reporting the prevalence of preconception care use explored the engagement with preconception care counselling services provided through community health clinics with a specific focus on women with type 1 or type 2 diabetes [56,61,63]. One large US population study (n = 6385) examined folic acid supplementation for women who had diagnosed diabetes or were classified as overweight or obese and found 35% of total respondents self-administered folic acid [52]. A Canadian study of women attending a health clinic (n = 464) found a notable difference in preconception care service use between women with type 1 (43.1%) and type 2 (18.4%) diabetes [56], a trend which was also reported in a similar UK study (n = 588) [61]. Within the subgroup of women with type 1 diabetes, those who: are younger; have lower weight; have longer duration of diabetes; and only use insulin for treatment, are more likely to report preconception care practices such as focusing on glucose control or family planning counselling [55].

### Women’s knowledge of preconception care benefits, preconception information sources and availability of services

This category overviews the findings from the included studies as it relates specifically to participants knowledge of the benefits of appropriate preconception care behaviours such as avoiding tobacco and alcohol use, undertaking regular exercise, or supplementing with folate. Women’s awareness of available preconception services and sources of information regarding preconception health are also included in this category. The examination of women’s knowledge of preconception care and availability of preconception services for women with a chronic health condition has been disparate and individual studies have explored the topic from different angles. However, significant gaps in women’s knowledge have been consistently identified across the six studies [53,54,57,63-65] examining this topic. Such gaps include a limited knowledge of the pregnancy risks associated with chronic medical conditions whether as a general risk [53,57,65], or associated with specific adverse outcomes [64]. A study involving focus groups of non-pregnant women with diabetes, hypertension or obesity also found that participants’ knowledge of behaviours which optimise preconception health and the impact of chronic health conditions on contraceptive choices was limited [53]. More than half (60%) of women who attended a hospital for antenatal services with diabetes were found to have no knowledge of the availability of a preconception service at the hospital.

A cross-sectional survey (n = 138) of women with type 1 diabetes investigating the factors that influence the women’s level of knowledge regarding pregnancy risk and preconception care found participants were better informed if they had a younger age of onset of the health condition, were multiparous, had a higher level of education, or monitored their blood glucose more than three times each day [64]. Sources of information related to pregnancy risk and preconception care needs for women with a chronic health condition were also explored. The majority of women (85%) had received information about pregnancy from a health professional or through leaflets, although 14% had received notification after a previous unplanned pregnancy and therefore were unable to engage with preconception services for that pregnancy [64]. This finding was also confirmed through a study drawing on semi-structured interviews of pregnant women with type 1 diabetes who sought advice from their usual health professional early in pregnancy with information only occasionally provided to them preconception [54]. Another smaller survey of women with type 1 diabetes (n = 27) complements the findings of the larger study but reported preferred rather than actual information sources. This smaller study indicated women with type 1 diabetes preferred their doctor or specialist as a source of information (81%) followed by books or leaflets (51%) the internet (46%) and diabetes clinic staff (46%). Standard media was the least preferred (30%) [65].

### Women’s attitudes and experiences of preconception care and preconception services

This category specifically relates to women’s attitudes towards the concept of using preconception services or the experiences of those who have used preconception services in the past and includes 6 identified studies [54,57-60,62]. Women reported experiencing emotional stress whilst attempting to comply with preconception requirements unique to their diagnosed health condition and avoid pregnancy complications [58,59,62,63], and needing discipline to follow the guidelines outlined by health professionals to minimise their pregnancy risk [62]. Others reported that women’s experience of preconception counselling contributed to fear of pregnancy complications to such a level that it made the decision to conceive more difficult [54]. Within the studies exploring women’s attitude and experience of preconception care, a trend towards fear and resistance to the present approach to preconception counselling including a perceived medicalisation of the condition [54,58,59] and a preference for more holism in the approach to preconception care [59] is evident.

Aside from these concerns, the decision to conceive is reportedly associated with a change in health behaviour, even for those women who were not always strict with their diet and lifestyle [62] which has been attributed to a desire to be as healthy as possible to ensure the best outcomes for the baby. However, this change is not always able to be maintained by the woman and can result in a haphazard approach to preconception care [58]. Barriers to women’s ability to engage with, or maintain, preconception practices not only include the emotional concerns highlighted above, but also employment type, particularly as they relate to the ability to implement and maintain diet and lifestyle change [62]. In addition, women have also identified the need to share private information, regarding their intentions to conceive, with employers to enable flexibility for medical appointments during work hours [58] and the perceived time needed for attending appointments [63]. This impact of time for appointments may also be exacerbated for those living in rural areas by the geographical distance to preconception service providers [63].

## Discussion

This review provides an examination of the prevalence and nature of the use of preconception care services by women with chronic health conditions worldwide. The significance of this work is underpinned by the international commitment to both preconception care and the management of chronic health in a population health context. Clear policy statements describing the significance and consequence of preconception care have been developed by international organisations and governing bodies [2-5]. A key element in these policies has been the importance of attenuating the severity of chronic health conditions prior to pregnancy to minimise the risk of obstetric and postnatal health problems. In line with this, a growing trend of research examining the use of preconception care practices by women with chronic health conditions is highlighted by this review, with 10 out of 14 studies published since 2010. As is common with the emerging nature of this research topic, there is inconsistency in some of the work conducted and limited scope in other areas. There are a number of gaps in available knowledge to inform those committed to translating the current policies and guidelines into meaningful and effective preconception programmes and services. One such gap is due to a limited approach to data collection and analysis which does not give adequate consideration to the needs of women with differing chronic health conditions, or the breadth and diversity of chronic health conditions which may benefit from preconception health services. Similarly, the scope of health behaviours and health services used by women with chronic health conditions for preconception care has been relatively narrow. Another knowledge gap in this field is due to a focus on retrospective cross-sectional studies with little examination of women’s change in use of health services prior to attempting to conceive, whilst actively planning conception, and into the antenatal period. Finally, very little of the existing research has examined the motivations, perceptions and attitudes towards preconception care and preferences for preconception services of women with chronic health conditions. Without dedicating future research attention to these areas, the development of preconception services and programs which are sensitive to the health behaviours and needs of the women they are targeting will be limited. As such, a possible agenda for future research to address these gaps, whilst not exhaustive, is proposed. Due to the breadth of research still needed in this field, the following agenda prioritises: sophistication in data analysis; longitudinal studies and improved representativeness; and attention to understanding the motivations, perceptions and attitudes of preconception care stakeholders.

### A broad and sophisticated approach to research design and analysis

There is value in broadening the range of chronic health conditions examined in the context of preconception care beyond the current dominant focus on type 1 diabetes. Given the known health implications of maternal type 2 diabetes [31], obesity [32], hypertension [33], depression [34], autoimmune disease [35] and thyroid disorders [37] for both mother and offspring, these additional conditions also require close attention. In fact, in light of the growing rates of obesity in both developed [67,68] and transitional [69,70] economies and the increasing awareness of the importance of addressing depression in contemporary maternity care [71] it could be argued that these two conditions need a priority focus in future research.

The development of knowledge in this topic area would also benefit from a differential approach to women’s conception intentions within research design and analysis. Current research indicates that women who are actively trying to conceive, those trying to avoid pregnancy, and those that are ambivalent differ in a range of characteristics such as age, parity, importance of motherhood, self-identifying a fertility problem, relationship satisfaction and economic hardship [72]. It may be beneficial to examine these groups of women separately and in contrast to one another given the potential differences in their preferences and needs from a preconception service, their ability to commit to preconception care planning or changes, and the goals which motivate them to make health behaviour changes. By taking this approach, researchers will be better positioned to provide meaningful findings to health professionals and policy makers which can inform the provision of tailored and relevant preconception care for all women of childbearing age with chronic health conditions.

Beyond a specific and discrete focus on women with chronic health conditions, there is also a need to expand the research scope to include other stakeholders such as women’s partners and health care providers. Given the impact a woman’s partner can have on women’s intention to conceive [72], and potentially on their engagement with preconception care [73], examination of the partners of women with a chronic health condition in the context of preconception care may provide valuable insights to assist in the development of policies and programmes. Likewise, the findings identified through this literature review suggest the current approach of health professionals providing preconception care is perceived as more medicalised and less holistic than would be preferred by women engaging with these professionals. Future research needs to more closely examine the interactions between health professionals and women with chronic health conditions undertaking preconception care to clarify this perception and assist health professionals in approaching these women in a manner which will encourage confidence and lasting health behaviour change.

A final avenue which would add breadth to our understanding of the preconception care patterns of women with chronic health conditions is the study of practices and behaviours women undertake as part of their preconception care. Current research examining health behaviours during the preconception period focus on smoking [20], alcohol use [21], diet [19] and exposure to communicable disease [22]. This work needs to be expanded to include focus upon general health service use such as visits with primary care practitioners including general practitioners and family physicians, but also specific health service use such as specialist doctors and complementary and alternative medicine (CAM) providers. In fact, given the high rates of CAM use reported for individuals with chronic health conditions [74,75], and the similarly high rates identified in women receiving fertility treatments [76], the use of CAM during the preconception period and for preconception care by women with chronic health conditions would benefit from focused attention.

### Improved consideration of temporal changes and sample representativeness

Current health services research which examines preconception care for women with chronic health conditions, with one exception [52], draws upon samples which are not generalisable due to the small sample sizes [53,57,58,63,65] and non-representative sampling methods [54-56,58-60,62-65]. As such the majority of work in this area has limited external validity. There is a clear need to explore this topic using nationally-representative samples to provide more robust insights into the prevalence of preconception health behaviours and patterns of preconception service use by women with chronic health conditions. A nationally-representative study design will enhance the external validity of any data in relationship to a target population of the country it is intended to represent [77]. In order for this research approach to best contribute to the current knowledge associated with the use of preconception care by women with chronic health conditions, it is important that such data includes a broad range of chronic health conditions reflective of the range of pre-pregnancy conditions linked with adverse birth outcomes, health behaviours, health service utilisation and attitudes towards health and health care. It would be most cost effective where possible to use existing data sources for this analysis (e.g. the Australian Longitudinal Study on Women’s Health linked to administrative data from the Medicare Benefits Schedule [78] for additional information on access to services).

Longitudinal data will also be a valuable component of future research as it allows for the examination of changes in behaviour throughout the childbearing years [79]. Drawing upon longitudinal data would enable researchers to explore differences in individual health behaviours, health service use, and attitudes towards health and health care during various life stages including long term preconception, immediate preconception, antenatal, postnatal and interconception periods. The use of longitudinal data would allow for not only comparisons across defined life stages but also changes for individual women over time. Within the context of chronic health conditions this level of analysis would identify the degree to which women with chronic health conditions change their behaviour once planning to conceive and also the degree to which these changes in behaviour continue on through pregnancy.

### The contribution of exploring motivations, attitudes and perceptions to understanding preconception practices

Complementing the above proposed research activities with a comprehensive examination of the motivations, attitudes and perceptions informing preconception health behaviour is vital. It is only through understanding these intrinsic characteristics of women with chronic health conditions that preconception programmes and services can be developed which respond adequately to women’s needs. Qualitative research designs are best placed to afford the necessary insights into this important aspect of preconception research. The approach of qualitative methodology is interpretive, pragmatic, grounded in the lived experiences of individuals, and commonly occurs in natural (rather than constructed) settings [80]. Application of research designs incorporating qualitative methods would enable an exploration of women’s perceived preferences and needs regarding preconception care service delivery including availability and access. This needs to be considered in the context of the current findings of emotional stress and fear but also the desire for holism within the preconception care services encountered by women with chronic health conditions identified within this review [54,58,59].

The use of qualitative methodology will also enable a closer examination of the motivations which may inform women using or not using preconception services, or modifying health behaviour in the preconception period – particularly for those intending to conceive. Similarly, it would provide an important opportunity to explore the perception and understanding of men regarding their role as male partners during the preconception care process for women with chronic health conditions, and likewise capture the experience and attitude of health professionals.

### Strength and limitations of this review

This review provides the first synthesis of health service research studies examining preconception care use by women with chronic health conditions. It highlights the limited evidence base currently available to guide policy and practice development. Most importantly it sets out a research agenda to expand the evidence base to enable better models of preconception care to be developed that respond to the needs of women with a range of chronic diseases.

This review has a number of limitations due to the heterogeneity of the studies restricting the ability to comprehensively compare findings across studies or interpret a consistent trend in results. Likewise, the dominance of small sample sizes utilised to provide the data reported in the studies restricts the capacity to infer implications from the findings beyond the study settings being examined. There was an over-representation of studies investigating type 1 diabetes in preference to other chronic health conditions and, as highlighted by one of the identified studies [56], the results from the research of type 1 diabetes may not represent the patterns and experiences of women with other chronic conditions. Similarly, the lack of differentiation between those intending to conceive and those who conceived accidently may confuse the results of the identified studies. Despite these limitations, this review provides the first overview of health services research associated with the topic globally and offers key insights for those providing care to women with chronic health conditions as well as offering a clear direction for the focus of future research on this topic.

## Conclusions

Preconception care for women with chronic health conditions is clearly a topic of increasing interest for researchers and of increasing importance for health care providers and policy makers. The health services research in this field is still emerging and even as the current evidence provides insights into the prevalence and knowledge of preconception care, attitudes towards preconception services and practices, and the characteristics of users for this subset of the population there remain significant research gaps requiring address. A framework for future research requires more sophisticated study design and methodology, the inclusion of more representative study populations, the analysis of longitudinal data, and dedicated attention to the attitudes, motivations and experiences of key stakeholders. By taking this more expansive and focused approach to the examination of preconception care for women with chronic health conditions, future research will be better placed to inform health professionals, health services managers and policy makers in the development of relevant and meaningful preconception care programs and services for women with chronic conditions.
